# Emotional resilience and sense of danger among doctors in hospitals during periods of heightened tensions and warfare in Israel

**DOI:** 10.1186/s13584-024-00655-3

**Published:** 2024-11-11

**Authors:** Michael V. Joachim, Dana Atia Joachim, Liat Korn, Yair Shapiro, Amir Laviv, Avi Zigdon

**Affiliations:** 1 Department of Health Systems Management, School of Health Sciences, Ariel University, Science Park, P.O.B. 3, Ariel, 40700 Israel; 2 Goldschleger School of Dentistry, Faculty of Medicine, Tel Aviv University, P.O.B. 39040, Tel Aviv, 6997801 Israel; 3 Unit of Oral and Maxillofacial Surgery, Shamir Medical Center, Tzrifin, Beer Yaacov, 7033001 Israel; 4 Unit of Oral and Maxillofacial Surgery, Samson Assuta Medical Center, Ha-Refu’a St 7, Ashdod, 7747629 Israel

**Keywords:** Conflict zones, Personal resilience, Sense of danger, Healthcare workers, Hospital security, Psychological impact, Iron swords war

## Abstract

**Background:**

The “Iron Swords” War beginning in October 2023 led to unprecedented levels of shock and trauma across Israel, significantly impacting the Israeli population and medical personnel. This study aimed to evaluate and compare the perceived personal resilience and sense of danger among physicians in hospitals located in different conflict zone proximities in Israel during this period.

**Methods:**

A quantitative, cross-sectional study was conducted from March to August 2024, during an active phase of the war, using a structured anonymous questionnaire. Participants were 161 physicians from three hospitals: one in southern Israel near the Gaza Strip, another in northern Israel near the borders with Lebanon, Syria, and Jordan, and a third in central Israel near Tel Aviv. The survey measured personal resilience using the Connor-Davidson Resilience Scale (CD-RISC-10) and sense of danger using the Solomon & Prager scale. Statistical analyses included Kruskal-Wallis H test, multiple linear regression, two-way analysis of variance (ANOVA), and Sobel test for mediation effects.

**Results:**

The final sample included 161 physicians (54 southern, 56 central, 51 northern). The mean resilience score was 31.14 ± 5.77, and the mean sense of danger score was 8.36 ± 4.15 (scales 0–40 and 0–20, respectively). Physicians in the southern hospital reported significantly higher sense of danger scores (*p* = 0.005). A trend towards lower resilience scores was noted among southern hospital physicians (*p* = 0.068) (*p* = 0.068). Two-way ANOVA revealed significant main effects of hospital location and gender on resilience (*p* = 0.046 and *p* = 0.003, respectively) and sense of danger (*p* = 0.005 and *p* = 0.062, respectively). Multiple regression analysis identified hospital location (β = -0.178, *p* = 0.023) and gender (β = 0.229, *p* = 0.004) as significant predictors of resilience. Mediation analysis indicated that personal resilience partially mediated the relationship between hospital location and sense of danger (indirect effect = 0.2896, *p* < 0.001).

**Conclusions:**

Physicians working near conflict zones report higher levels of perceived danger, though their resilience is comparable to peers in less threatened regions. Enhancing personal resilience is crucial to mitigate the heightened sense of danger. This could include regular resilience training, psychological support, and specific programs for single and childless physicians to improve safety perceptions. Additionally, fostering a supportive community with clear communication and robust emergency protocols is essential for enhancing staff resilience and safety in hospitals.

## Background

Throughout its history, Israel faced a series of significant security challenges, often requiring swift transitions between states of normalcy and emergency. However, the “Iron Swords” War that erupted in October 2023 marked an unprecedented level of shock and trauma within the Israeli population. The profound impact of this tragedy permeated all segments of society, transcending traditional political divisions. Several research studies have documented sharp increases in anxiety-related symptoms, including uncontrolled fear, physiological hyper-arousal, and insomnia since the beginning of the “Iron Swords” war [1, 2].

During periods of heightened tensions and warfare, medical facilities located near active conflict zones face formidable challenges [3, 4]. In addition to their primary responsibility of providing immediate medical care to the injured, these facilities must navigate between significant security threats, including rocket attacks, terrorist infiltrations, and other hazards associated with conflict zones [5, 6]. The effective performance of medical personnel under such conditions necessitates robust leadership, active management involvement and clear emergency guidelines to ensure optimal functioning of healthcare services under extreme conditions [7, 8]. Beyond treating physical injuries and managing psychological trauma, such as shell shock, medical personnel are tasked with the immense responsibility of ensuring their own safety while addressing the overwhelming needs of their patients. Hospitals situated on the front lines became battlefields, often overwhelmed by a surge of wounded individuals. In these scenarios, various factors can severely impact the ability of medical teams to attend work [9–11]. Research conducted in Israel indicated that health care workers are less inclined to report for duty during times of terrorist threats [12]. Globally, attendance among healthcare workers tends to be higher following natural disasters and epidemics exceeding 45% but drops significantly in response to events such as mass shootings, terrorist threats and biological warfare falling below 37%, largely due to diminished confidence and concerns over personal and family safety [11].

During emergencies, the reluctance of medical staff, particularly physicians, to report for duty or their challenges in performing effectively can often be attributed to family responsibilities, such as caring for children and the older adult, as well as the stress and burnout associated with prolonged and intensive work periods [13]. Personal and family security has consistently been highlighted as a fundamental need, often taking precedence over professional obligations [14, 15]. As the level of danger increases, ensuring the safety of oneself and one’s family becomes paramount for medical personnel. Consequently, healthcare institutions must prioritize addressing these security concerns to maintain staff commitment and effectiveness during emergencies [16, 17]. Previous research has demonstrated that an increased sense of danger and concerns about potential threats are associated with low mental resilience, inadequate coping skills, and elevated stress levels [5, 18]. Moreover, studies conducted in Israel have identified a significantly heightened sense of danger among residents in the southern region, particularly within 40 km from the Gaza Strip [19].

Resilience is understood both as process and as an outcome of successfully adapting to difficult or challenging life experiences, primarily facilitated by mental, emotional, and behavioral flexibility in response to external and internal demands [20–23]. Community resilience, in turn, refers to the collective capacity of a community to recover from a challenging event and rapidly return to a state of normality. In contrast, national resilience encompasses society’s overall vitality and adaptability across various domains when facing challenges [5, 19, 23, 24]. Personal resilience is an individual’s capacity to effectively navigate challenging events, such as disasters or wars, and restore their previous functioning level in the shortest possible time [25, 26]. Therefore, the ability to perform effectively in stressful work situations is directly and significantly influenced by workers’ personal resilience [27]. Previous studies have consistently demonstrated a positive correlation between resilience at the national, community, and personal levels [28, 29]. Despite the State of Israel frequently encountering numerous emergency situations, the resilience of its medical teams remains notably lower compared to their counterparts in the United States [30, 31]. Potential factors contributing to this include reduced personal security, inadequate support systems for children during danger, and the perceived risks associated with hospital commutes [5, 30, 31]. The observed results contradict the expected enhancement in mental resilience among medical teams with experience handling emergency situations, which is noteworthy given Israel’s substantial experience in dealing with sudden emergencies [32, 33].

The attack on October 7th, 2024, on Israel marked one of most challenging and traumatic events, leading to the onset of the “Iron Swords” war. This war has significantly affected the overall sense of security in various regions and required intensive efforts from healthcare professionals across the entire healthcare system [34]. The impact, however, was asymmetrical. Hospitals in the southern region bore the brunt of the patient influx and prolonged missile danger [35, 36], whereas those in the northern region faced threats to a lesser extent and received fewer wounded patients. In contrast, medical centers in the central region served as a second line for treating combat zone casualties, experiencing an increased workload and remaining vulnerable to missile attacks [37]. This study aimed to evaluate and compare the perceived personal resilience and sense of danger among physicians in hospitals located in different conflict zone proximities in Israel during this period. It placed particular emphasis on examining how levels of personal security differ with the hospital’s proximity to conflict zones, and on assessing how personal resilience and sense of danger are influenced by demographic factors, such as age, marital status, and the presence and ages of children.

## Materials and methods

### Study design

This study employed a quantitative, retrospective, cross-sectional design, utilizing a structured and anonymous questionnaire. Data were collected between March and August 2024 from three public hospitals in Israel, comprising a total of 161 physicians (54, 56 and 51 physicians from the 3 hospitals). The first hospital, located in southern Israel, approximately 25 km from the Gaza Strip, is in close proximity to conflict zones and under near-constant aerial threat. The second hospital, situated in northern Israel, 30–40 km from the borders with Lebanon, Syria, and Jordan, while not in direct proximity to active combat zones, is subject to some degree of aerial threat. The third hospital is centrally positioned near Tel Aviv’s international airport in central Israel, an area that has experienced relatively few past aerial threat alarms.

The study was conducted during an active phase of the “Iron Swords” war, reflecting the physicians’ reported perceptions during that time. All medical centers had specialized fortified wards, including operating rooms, that remained fully operational during the period.

### Ethical considerations

The study received ethical approval from the Ariel University ethical committee (ref AU-HEA-AZ-20240317). Written informed consent was obtained from all participants prior to completing the questionnaire. Questionnaires were coded for anonymous data analysis.

### Participants

The study included participants 161 physicians from three public hospitals in Israel, strategically chosen based on their geographical contexts. a southern hospital near the Gaza Strip (54 participants), a central hospital near Tel Aviv (56 participants), and a northern hospital near the borders with Lebanon, Syria, and Jordan (51 participants). The target population comprised physicians who met the following criteria: hospital staff during October 2023 who were actively participating in patient treatment. Exclusion criteria were non-physicians, outsourced physicians, and refusal to complete the questionnaire. Participant recruitment utilized a dual approach. Firstly, the researchers personally reached out to potential participants, extending invitations to take part in the study. Additionally, the existing participants were asked to recommend other eligible individuals from their professional networks, who were then approached and invited to participate as well. The survey was sent via email or personal phone messages, and respondents were asked to voluntarily complete an online survey.

### Measures

The online questionnaires examined perceived personal resilience and reported sense of danger. All participants provided comprehensive socio-demographic and professional information. This included their age, gender, and marital status (categorized as single, married/partnered, or divorced/widowed). Information about family structure was collected, including whether the participant had children and, if so, the ages of their children (grouped into categories of under 2 years, 2–10 years, 10–18 years, and over 18 years). Participants also reported their religious affiliation, choosing from options including Jewish, Muslim, Christian, Druze, or other.

Professional information gathered included the participant’s status (intern, resident, or senior physician), their specific medical specialty, and years of professional experience. The location of their hospital (southern, central, or northern) was also recorded.

Individual Resilience was measured using the abridged Hebrew version of the Connor-Davidson Resilience Scale (CD-RISC-10), validated by Campbell & Stein [38]. The CD-RISC-10 consists of 10 statements describing different resilience aspects (flexibility, self-efficacy, ability to regulate emotion, optimism, and maintaining attention under stress), rated on a 5-point scale from 0 (not at all true) to 4 (true nearly all the time). The total score ranges from 0 to 40, with higher scores suggesting greater resilience and lower scores indicating less resilience or difficulty bouncing back from adversity [39]. In the original validation study, the CD-RISC-10 demonstrated good internal consistency with a Cronbach’s alpha of 0.85 [38]. In the current study, the Cronbach’s alpha was found to be α = 0.869, indicating high reliability.

Sense of Danger was evaluated using the Solomon & Prager Sense of Danger scale, measuring personal, family, workplace, and homeland aspects of danger [24, 40]. The items were rated on a scale from 0 (not true at all) to 4 (almost always true). The total score ranges from 0 to 20, with higher scores suggesting higher sense of danger. In previous studies, the Sense of Danger scale demonstrated good internal consistency with Cronbach’s alpha values ranging from 0.80 to 0.85 [24, 40]. In the current study, the Cronbach’s alpha was found to be α = 0.859, indicating high reliability.

### Data analysis

Statistical analyses were conducted using GraphPad Software Inc. (La Jolla, CA) and IBM SPSS Statistics (Version 27, Armonk, NY). Continuous variables are presented as the mean ± standard deviation (SD), while dichotomous and categorical variables are presented as frequencies and percentages. Parametric tests were employed for normally distributed continuous variables, including the independent t-test for comparing means between two groups and one-way Analysis of Variance (ANOVA) for comparisons across more than two groups. Two-way ANOVA was used to examine the effects of two independent variables and their interaction on continuous outcomes. Simple linear regression was used to examine relationships between continuous predictors and outcomes, while multiple linear regression was employed to assess the combined effects of multiple predictors on outcomes. Non-parametric tests were utilized for non-normally distributed variables or ordinal data. The Kruskal-Wallis H test was used to compare medians across multiple groups, and the Mann-Whitney U test was applied for comparisons between two groups. The Chi-square test was used for analyzing relationships between categorical variables. For the analysis of mediation effects, multiple linear regression was performed to evaluate the direct and indirect effects, with a Sobel test to assess the significance of the indirect effect through the proposed mediator. All statistical tests were two-tailed, and statistical significance was defined as *p* < 0.05, with trends noted for *p* < 0.10.

## Results

Data were collected from March to August 2024 across three public hospitals in Israel. The final sample consisted of 161 physicians, with 54 from the Southern Hospital, 56 from the Central Hospital, and 51 from the Northern Hospital.

Participants ranged in age from 25 to 65 years, with a mean age of 41.85 ± 9.37 years. Among the participants, 96 (59.6%) were male, 115 (71.4%) were married or lived with a partner, and 123 (76.4%) had children. Of those with children, 90 (55.9%) had children under 10 years old. The majority (98, 60.9%) were senior physicians, including managers and chairs. Religious affiliation data shows that 121 (75.2%) belonged to the Jewish community, with the remainder distributed among Muslim, Christian, Druze, and other affiliations. Table 1 presents the detailed demographic characteristics.

Overall, there were no significant differences between the respondent groups in the 3 hospitals in terms of age (*p* = 0.501), gender (*p* = 0.743), marital status (*p* = 0.188), presence and ages of children (*p* = 0.116), and hospital position (*p* = 0.479).

**Table 1:** Demographic characteristics of the respondents

Variable		South(*n* = 54) n (%)	Center (*n* = 56) n (%)	North (*n* = 51) n (%)	*p* value ($\:{\varvec{\chi\:}}^{2}$)	Total (*N* = 104) n(%)
Age (mean 41.85 ± 9.37)	25–40	27 (50.0)	24 (42.9)	28 (54.9)	0.501 (1.378)	79 (49.1)
	41–65	27 (50.0)	32 (57.1)	23 (45.1)		82 (50.9)
Gender	Male	34 (63.0)	33 (58.9)	29 (56.9)	0.743 (0.594)	96 (59.6)
	Female	20 (37.0)	23 (41.1)	22 (43.1)		65 (40.4)
Family Status	Single	11 (20.4)	9 (16.1)	9 (17.6)	0.188 (6.150)	29 (18.0)
	Married / Partner	37 (68.5)	43 (76.8)	35 (68.6)		115 (71.4)
	Divorced / Widowed	6 (11.1)	4 (7.1)	7 (13.7)		17 (10.6)
Hospital Position	Intern	5 (9.3)	6 (10.7)	8 (15.7)	0.479 (3.498)	19 (11.8)
	Resident	17 (31.5)	15 (26.8)	12 (23.5)		44 (27.3)
	Senior	32 (59.3)	35 (62.5)	31 (60.8)		98 (60.9)
Children’s age (yrs)	None	14 (25.9)	12 (21.4)	12 (23.5)	0.116 (13.102)	38 (23.6)
	< 2	6 (11.1)	7 (12.5)	10 (19.6)		23 (14.3)
	2–10	24 (44.4)	27 (48.2)	16 (31.4)		67 (41.6)
	10–18	15 (27.8)	17 (30.4)	8 (15.7)		40 (24.8)
	> 18	10 (18.5)	12 (21.4)	11 (21.6)		33 (20.5)
	Total	69	75	57		201

The analyzing of age as a continuous variable showed no significant difference in mean age between the hospitals (*p* = 0.223, ANOVA F-ratio = 1.514). The overall mean age was 41.85 ± 9.37 years.

The mean resilience score on the CD-RISC-10 was 31.14 ± 5.77. The tests showed that the CD-RISC-10 scores and the scores for the perception of danger were not normally distributed (Shapiro-Wilk test for CD-RISC-10 scores: W(161) = 0.97, *p* = 0.002, and for sense of danger score: W(161) = 0.97, *p* = 0.001 showed a significant deviation from the normal distribution). When analyzing the results between the three medical centers, there was a trend towards a significant difference in the Kruskal-Wallis H test for CD-RISC-10 scores (χ2(2) = 5.37, *p* = 0.068). The mean sense of danger score was 8.36 ± 4.15. Analyzing the results between the three medical centers revealed a significant difference in the Kruskal-Wallis H test for sense of danger scores (χ2(2) = 10.76, *p* = 0.005). Overall comparisons between medical centers in CD-RISC-10 and Sense of danger scores are shown in Table 2.

**Table 2:** CD-RISC-10 and sense of danger questionnaire results (IQR – interquartile range)

Variable		South (*n* = 54)	Center (*n* = 56)	North (*n* = 51)	*p* value (Kruskal Wallis H)
CD-RISC-10 score (mean 31.2 ± 5.88)	Mean ± SD	29.80 ± 6.29	31.96 ± 5.11	31.69 ± 5.70	
	Median	31	32	32	0.068 (5.371)
	IQR	9	7	8	
Sense of danger score (mean 7.83 ± 4.2)	Mean ± SD	9.13 ± 3.76	7.18 ± 3.93	8.84 ± 4.56	
	Median	9	7	8	0.005 (10.763)
	IQR	5	6	7	

**Fig. 1:**
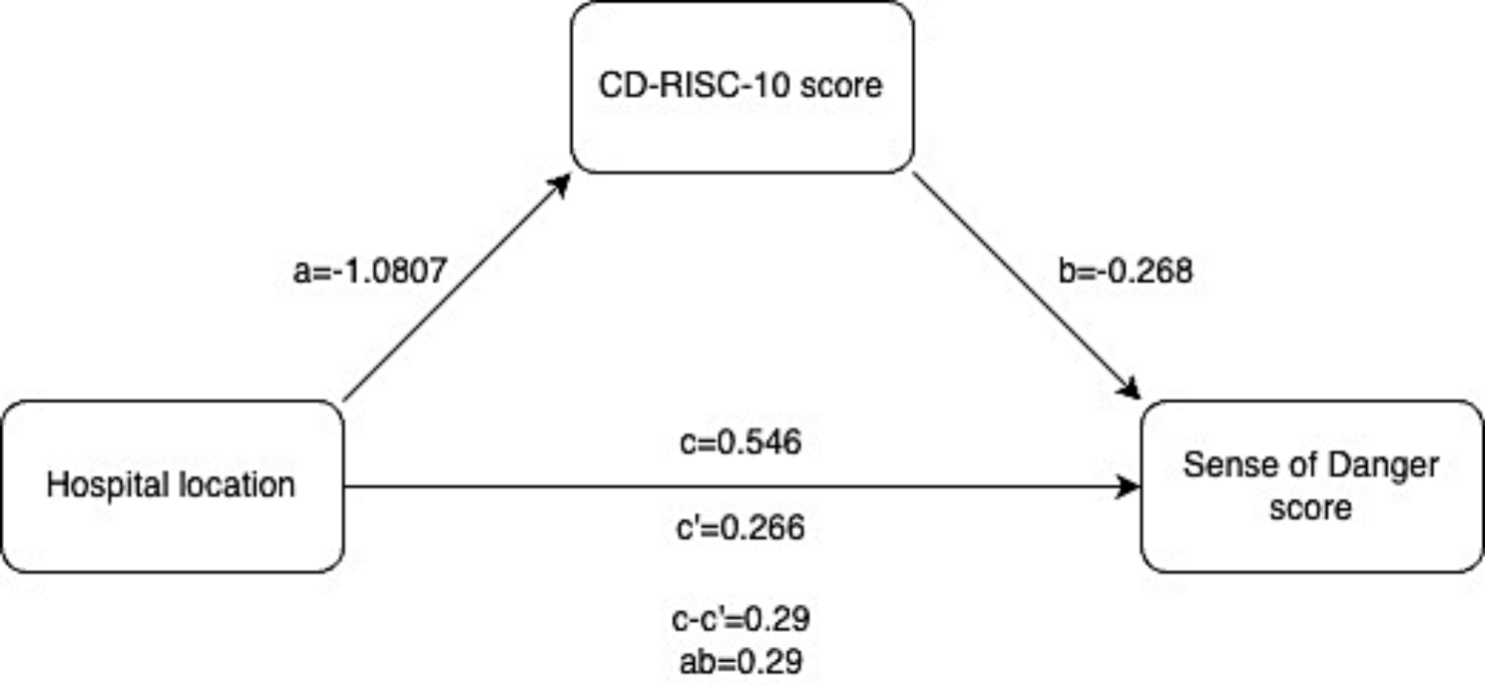
Mediation model showing the relationships among hospital location, CD-RISC-10 score (mediator), and Sense of Danger score. Path coefficients are provided for the direct effects (c’ and b), the total effect (**c**), and the indirect effect (**a** × **b**). The results indicate partial mediation, where CD-RISC-10 score partially explains the relationship between hospital location and Sense of Danger score

Next, we checked whether the CD-RISC-10 score mediates the relationship between hospital location and sense of danger score. The results from the regression analyses and the Sobel test revealed a significant indirect effect, providing evidence for partial mediation. Specifically, the indirect effect of hospital location on sense of danger score through CD-RISC-10 score was statistically significant (indirect effect = 0.2896, 95% CI: 0.1843, 0.3949). Furthermore, the bootstrapping analysis confirmed these findings, with approximately 53% of the total effect of hospital location on sense of danger score being mediated by CD-RISC-10 score (PM = 0.5301). While the direct effect of hospital location on sense of danger score remained significant after accounting for the mediator (path c’ = 0.2566, *p* = 0.012), its magnitude was reduced compared to the total effect (path c = 0.5462), indicating partial mediation. These results suggest that an individual’s level of resilience partially explains the relationship between hospital location and perceived sense of danger. The mediation model results are presented in Fig. 1.

Analysis revealed a significantly higher resilience score among males (*p* = 0.002, Mann Whitney U test), while there was a trend towards a significant relation to gender on the sense of danger (*p* = 0.067, Mann Whitney U test), with females reporting higher levels. The results are presented in Table 3.

**Table 3:** Distribution of CD-RISC-10 and sense of danger mean scores by gender

Variable		Male (= 63)	Female (*n* = 41)	*p* value (Mann Whitney U test Z score)
CD-RISC-10 score	Mean	32.24	29.55	0.002 (3.073)
	SD	5.78	5.57	
Sense of danger score	Mean	7.88	9.06	0.067 (-1.832)
	SD	3.97	4.34	

A two-way ANOVA was conducted to examine the effects of hospital location and gender on CD-RISC-10 and Sense of Danger scores. For CD-RISC-10 scores, significant main effects were found for both hospital location (F(2, 155) = 3.15, *p* = 0.046) and gender (F(1, 155) = 9.24, *p* = 0.003), but no significant interaction effect was observed (F(2, 155) = 0.78, *p* = 0.460). For Sense of Danger scores, a significant main effect was found for hospital location (F(2, 155) = 5.42, *p* = 0.005), a trend towards significance for gender (F(1, 155) = 3.52, *p* = 0.062), and no significant interaction effect (F(2, 155) = 0.34, *p* = 0.713). These results suggest that both hospital location and gender independently influence resilience and sense of danger, but their effects do not significantly interact.

Analysis of marital status showed no significant influence on resilience score (*p* = 0.539, Mann Whitney U test), and no significant difference in the sense of danger, although single/divorced/widowed participants reported slightly higher levels (*p* = 0.183, Mann Whitney U test). The results are presented in Table 4.

**Table 4:** Marital status on CD-RISC-10 and sense of danger scores

Variable		Married / Partner (*N* = 77)	Single / Divorced / Widowed (*N* = 27)	*p* value (Mann Whitney U test Z score)
CD-RISC-10 score	Mean	31.30	30.74	0.539 (-0.614)
	SD	5.62	6.17	
Sense of danger score	Mean	8.10	9.00	0.183 (-1.332)
	SD	4.07	4.30	

The presence of children had no significant effect on the resilience score (*p* = 0.418, Mann Whitney U test) or sense of danger (*p* = 0.912, Mann Whitney U test) in the overall sample. A specific analysis at the southern hospital revealed a significantly higher sense of danger for the childless group (*p* = 0.038, Mann Whitney U test), while no significant results were found for the north (*p* = 0.917) or center (*p* = 0.650). No significant differences were found in resilience scores between those with and without children at any of the hospitals (South: *p* = 0.779, Center: *p* = 0.368, North: *p* = 0.683).

Multiple regression analyses were conducted to examine the relative influence of various factors on CD-RISC-10 and Sense of Danger scores. For CD-RISC-10 scores (Table 5), the model explained 12.4% of the variance (R² = 0.124, F(5, 155) = 4.38, *p* < 0.001). Hospital location (β = -0.178, *p* = 0.023) and gender (β = 0.229, *p* = 0.004) were significant predictors, with males and those in hospitals further from conflict zones showing higher resilience. For Sense of Danger scores (Table 6), the model explained 10.8% of the variance (R² = 0.108, F(5, 155) = 3.75, *p* = 0.003). While the overall model was significant, no individual predictor reached statistical significance, although gender showed a trend (β = -0.140, *p* = 0.073), with females tending to report higher sense of danger.

**Table 5:** Multiple regression analysis for factors predicting CD-RISC-10 scores

Predictor	B	SE	β	t	*p*
Hospital Location	-1.081	0.472	-0.178	-2.291	0.023
Gender (Male)	2.691	0.912	0.229	2.951	0.004
Age	0.052	0.049	0.084	1.061	0.290
Marital Status (Married)	0.556	1.059	0.044	0.525	0.600
Having Children	-0.173	1.123	-0.013	-0.154	0.878

Note: B = unstandardized regression coefficient; SE = standard error; β = standardized regression coefficient; t = t-statistic; p = p-value. R² = 0.124, F(5, 155) = 4.38, *p* < 0.001

**Table 6:** Multiple regression analysis for factors predicting sense of danger scores

Predictor	B	SE	β	t	*p*
Hospital Location	0.546	0.338	0.126	1.616	0.108
Gender (Male)	-1.180	0.653	-0.140	-1.807	0.073
Age	-0.033	0.035	-0.075	-0.943	0.347
Marital Status (Married)	-0.890	0.758	-0.098	-1.174	0.242
Having Children	0.205	0.804	0.022	0.255	0.799

Note: B = unstandardized regression coefficient; SE = standard error; β = standardized regression coefficient; t = t-statistic; p = p-value. R² = 0.108, F(5, 155) = 3.75, *p* = 0.003

## Discussion

This study aimed to evaluate and compare the perceived personal resilience and sense of danger among physicians in hospitals located in different conflict zone proximities in Israel during this period. Our findings paint a vivid picture of the psychological landscape faced by healthcare professionals in a nation at war, revealing both expected patterns and surprising resilience in the face of unprecedented challenges. They also reveal significant regional differences in the sense of danger experienced by medical staff, with physicians at the Southern Hospital, situated near the Gaza Strip, reporting the highest levels of perceived danger. Conversely, physicians at the hospital in central Israel reported the lowest sense of danger. This gradient of perceived threat highlights the relationship between conflict proximity and the psychological well-being of healthcare workers [5, 41].

The elevated sense of danger observed among physicians at the southern hospital aligns with previous research findings that documented a higher sense of danger in southern regions of Israel, particularly within a 40 km radius of the Gaza Strip [2, 19]. The heightened sense of danger is linked to the hospital’s closeness to conflict zones and the risk of missile attacks or other threats [5, 12]. However, it’s important to note that hospitals in the northern region are also exposed to potential threats due to their proximity to borders with Lebanon, Syria, and Jordan. The stark contrast in perceived danger between these regions illuminates the multifaceted nature of threat perception, which extends beyond mere geographical proximity to conflict zones. The difference in perceived danger between these regions may be attributed to various factors beyond mere proximity to borders. These could include the nature and frequency of past security incidents, the intensity of the current conflict in each area, media coverage of threats, and public perception of risk. For instance, the southern region’s experience with frequent rocket attacks and the recent unprecedented ground incursion may contribute to a heightened sense of danger compared to other areas, despite the presence of potential threats in the north as well [42, 43].

The implications of these regional disparities in perceived danger are far-reaching and multifaceted, touching every aspect of healthcare delivery and workforce management. In areas with higher perceived danger, such as the southern region, healthcare systems may face challenges in staff retention and recruitment [44]. This could lead to understaffing or higher turnover rates, potentially compromising the quality and continuity of care. Moreover, the heightened sense of danger might affect decision-making processes and risk assessments made by healthcare professionals, potentially influencing treatment plans and resource allocation [3]. Healthcare managers in these high-risk areas may need to implement more robust support systems, including enhanced security measures, regular psychological support, and clear emergency protocols, to mitigate the impact of perceived danger on staff performance and wellbeing [36, 45, 46].

One of the most intriguing findings of our study is the lack of significant difference in resilience scores across hospitals, which challenges our initial hypotheses and reveals the complex nature of psychological resilience in high-stress environments. This unexpected result may reflect the multifaceted nature of resilience itself, which is influenced by various personal, professional, and environmental factors [47]. The “Iron Swords” war presented unprecedented challenges across all regions of Israel, potentially affecting healthcare workers’ resilience regardless of their specific location. This widespread impact might have led to a more uniform resilience response across different areas, suggesting that the human capacity for adaptation may be more flexible and robust than previously thought [5, 41, 48].

Additionally, the professional nature of our sample - all participants being physicians - may have played a role. Physicians, as a group, often demonstrate high baseline levels of resilience due to their training and regular exposure to high-stress situations [49]. This inherent resilience, honed through years of medical training and practice, may serve as a psychological buffer against the acute stresses of conflict, creating a “ceiling effect” that makes it more challenging to detect location-based differences in resilience [50, 51].

It’s also worth considering that the relationship between proximity to conflict and resilience may not be linear, but rather follow a more complex, even counterintuitive pattern. While closer proximity to conflict zones might intuitively seem to decrease resilience, it could also foster adaptive coping mechanisms and strengthen resilience over time [52]. This phenomenon, sometimes called “stress inoculation” or “adversity-activated development,” suggests controlled exposure to stress can enhance one’s ability to cope with future challenges [25, 26, 52]. This complex interaction between adversity and resilience development may have contributed to the observed non-significant differences.

Healthcare managers in these high-risk areas may need to implement more robust support systems, including enhanced security measures, regular psychological support, and clear emergency protocols, to mitigate the impact of perceived danger on staff performance and wellbeing [32, 33]. The observed differences can be attributed to the specific adversities presented by the “Iron Swords” conflict, which stands apart from earlier skirmishes due to its heightened severity, extended timeframe, and the broad-scale peril of missile barrages throughout the nation, amid persistent security issues.

Our mediation analysis revealed that personal resilience partially mediated the relationship between hospital location and sense of danger. This finding aligns with previous studies highlighting the protective role of resilience in mitigating the impact of adverse or stressful circumstances on psychological well-being [53–55]. Partial mediation suggests that while resilience plays a crucial role in buffering the effects of location-based stressors, other factors related to hospital location also contribute to an individual’s perceived sense of danger. This complex interplay between resilience, location, and perceived danger underscores the need for multifaceted interventions to support healthcare workers in high-risk areas.

The analysis of demographic factors provided additional insights into the dynamics of resilience and perceived danger among physicians. Single, divorced, or widowed physicians reported a borderline significantly higher sense of danger compared to their married or partnered counterparts. This finding aligns with Maslow’s hierarchy of needs theory, emphasizing the fundamental nature of personal and family security [14, 15]. The absence of an immediate family support system may create a psychological vacuum that amplifies perceived threats, underscoring the importance of social support networks in maintaining psychological well-being during times of crisis [56].

One of the most surprising findings of our study was the localized effect of childlessness on perceived danger, particularly in the southern hospital. While the presence of children did not significantly impact resilience scores or the sense of danger among physicians overall, childless physicians in the southern hospital reported a significantly higher sense of danger compared to those with children. This localized effect presents a fascinating paradox: in the area of highest objective danger, having children appears to serve as a psychological buffer rather than an additional source of worry. This counterintuitive finding might be explained by the additional perceived vulnerability and lack of immediate familial obligations among childless physicians, potentially shifting the focus more toward personal safety concerns in high-risk areas. Additionally, the protective effect of having children in high-risk areas may be related to increased resilience due to social connections and sense of purpose [57].

Gender differences in resilience scores, with male physicians exhibiting higher resilience compared to their female counterparts, align with previous research [58, 59]. This gender disparity in resilience scores opens up important questions about the interplay between societal expectations, professional roles, and psychological coping mechanisms in high-stress environments. This observation may be attributed to a combination of cultural, societal, and biological factors, including traditional gender roles, expectations, and differences in stress response mechanisms [45, 46]. The finding that women reported more anxiety symptoms during the acute phase of the “Iron Swords” war further supports this gender-based disparity in psychological responses to conflict situations [1].

The lack of significant correlation between age and resilience or sense of danger scores across the entire sample is consistent with previous CD-RISC-based research [38, 60, 61]. This age-independent resilience challenges common assumptions about the relationship between life experience and psychological hardiness, suggesting that the ability to withstand stress may be more related to individual traits and training than to years lived. However, this finding should be interpreted cautiously, considering the unique characteristics of the study population. Physicians, regardless of age, are often exposed to high-stress situations and may develop resilience early in their careers, potentially masking age-related differences that might be observed in the general population.

These findings underscore the importance of tailored interventions to support healthcare professionals in different regions based on the specific risks and challenges they face. Enhancing resilience among medical staff, particularly in high-threat areas like the southern region, is crucial [62]. Targeted strategies could include regular resilience training programs that address the unique stressors faced by healthcare workers in conflict zones, as well as psychological support services tailored to the needs of different demographic groups, including single and childless physicians. Implementing robust security measures and clear emergency protocols is essential to ensure a safer working environment and boost confidence among staff [2, 36, 63–65]. Creating supportive communities within hospitals, fostering open communication, and establishing peer support networks can further enhance resilience [65, 66]. Regular drills and simulations can reinforce emergency preparedness and build collective resilience [67]. These interventions should be designed with consideration for gender differences in resilience and coping mechanisms, as well as the unique challenges faced by physicians in different family situations and career stages [45, 46, 58, 59]. By implementing these strategies, can protect staff well-being while ensuring continuity and quality of care during challenging circumstances. The resilience demonstrated by physicians in this study serves as a testament to the human capacity for adaptation and perseverance in the face of adversity, offering valuable lessons for healthcare systems worldwide grappling with crises and conflicts.

### Limitations

This study has several limitations that should be considered when interpreting its results. The relatively small sample size and focus on three hospitals restrict the generalizability of our findings. This limitation may explain the lack of statistically significant differences in resilience scores across hospital locations, despite observed trends. Despite efforts to mitigate potential biases associated with convenience sampling through measures such as diverse specialty representation, balanced recruitment, and statistical adjustments, the possibility of residual selection bias cannot be eliminated. This may limit the extrapolation of our findings to the broader population of physicians in Israel.

## Conclusions

Physicians working near conflict zones report higher levels of perceived danger, though their resilience is comparable to peers in less threatened regions. To mitigate the heightened sense of danger, it is crucial to enhance personal resilience through targeted support strategies. This could include resilience training, psychological support, and targeted programs for single and childless physicians to enhance safety perceptions. Additionally, fostering a supportive community with clear communication and robust emergency protocols is essential for enhancing staff resilience and safety in hospitals.

## Data Availability

The data used in this study is available from the authors. However, Ariel University’s research ethics committee’s and the School of Health Sciences’ approvals are required upon reasonable request.

## Abbreviations

CD-RISC-10: Connor-Davidson Resilience Scale (10-item version)
PTSD: Post-Traumatic Stress Disorder
SoD: Sense of Danger
IDF: Israel Defense Forces
WHO: World Health Organization
SPSS: Statistical Package for the Social Sciences
